# Author Correction: National identity predicts public health support during a global pandemic

**DOI:** 10.1038/s41467-022-29658-x

**Published:** 2022-04-06

**Authors:** Jay J. Van Bavel, Aleksandra Cichocka, Valerio Capraro, Hallgeir Sjåstad, John B. Nezlek, Tomislav Pavlović, Mark Alfano, Michele J. Gelfand, Flavio Azevedo, Michèle D. Birtel, Aleksandra Cislak, Patricia L. Lockwood, Robert Malcolm Ross, Koen Abts, Elena Agadullina, John Jamir Benzon Aruta, Sahba Nomvula Besharati, Alexander Bor, Becky L. Choma, Charles David Crabtree, William A. Cunningham, Koustav De, Waqas Ejaz, Christian T. Elbaek, Andrej Findor, Daniel Flichtentrei, Renata Franc, Biljana Gjoneska, June Gruber, Estrella Gualda, Yusaku Horiuchi, Toan Luu Duc Huynh, Agustin Ibanez, Mostak Ahamed Imran, Jacob Israelashvili, Katarzyna Jasko, Jaroslaw Kantorowicz, Elena Kantorowicz-Reznichenko, André Krouwel, Michael Laakasuo, Claus Lamm, Caroline Leygue, Ming-Jen Lin, Mohammad Sabbir Mansoor, Antoine Marie, Lewend Mayiwar, Honorata Mazepus, Cillian McHugh, John Paul Minda, Panagiotis Mitkidis, Andreas Olsson, Tobias Otterbring, Dominic J. Packer, Anat Perry, Michael Bang Petersen, Arathy Puthillam, Julián C. Riaño-Moreno, Tobias Rothmund, Hernando Santamaría-García, Petra C. Schmid, Drozdstoy Stoyanov, Shruti Tewari, Bojan Todosijević, Manos Tsakiris, Hans H. Tung, Radu G. Umbreș, Edmunds Vanags, Madalina Vlasceanu, Andrew Vonasch, Meltem Yucel, Yucheng Zhang, Mohcine Abad, Eli Adler, Narin Akrawi, Hamza Alaoui Mdarhri, Hanane Amara, David M. Amodio, Benedict G. Antazo, Matthew Apps, F. Ceren Ay, Mouhamadou Hady Ba, Sergio Barbosa, Brock Bastian, Anton Berg, Maria P. Bernal-Zárate, Michael Bernstein, Michał Białek, Ennio Bilancini, Natalia Bogatyreva, Leonardo Boncinelli, Jonathan E. Booth, Sylvie Borau, Ondrej Buchel, C. Daryl Cameron, Chrissie F. Carvalho, Tatiana Celadin, Chiara Cerami, Hom Nath Chalise, Xiaojun Cheng, Luca Cian, Kate Cockcroft, Jane Conway, Mateo Andres Córdoba-Delgado, Chiara Crespi, Marie Crouzevialle, Jo Cutler, Marzena Cypryańska, Justyna Dabrowska, Michael A. Daniels, Victoria H. Davis, Pamala N. Dayley, Sylvain Delouvee, Ognjan Denkovski, Guillaume Dezecache, Nathan A. Dhaliwal, Alelie B. Diato, Roberto Di Paolo, Marianna Drosinou, Uwe Dulleck, Jānis Ekmanis, Arhan S. Ertan, Tom W. Etienne, Hapsa Hossain Farhana, Fahima Farkhari, Harry Farmer, Ali Fenwick, Kristijan Fidanovski, Terry Flew, Shona Fraser, Raymond Boadi Frempong, Jonathan A. Fugelsang, Jessica Gale, E. Begoña Garcia-Navarro, Prasad Garladinne, Oussama Ghajjou, Theofilos Gkinopoulos, Kurt Gray, Siobhán M. Griffin, Bjarki Gronfeldt, Mert Gümren, Ranju Lama Gurung, Eran Halperin, Elizabeth Harris, Volo Herzon, Matej Hruška, Guanxiong Huang, Matthias F. C. Hudecek, Ozan Isler, Simon Jangard, Frederik J. Jørgensen, Frank Kachanoff, John Kahn, Apsara Katuwal Dangol, Oleksandra Keudel, Lina Koppel, Mika Koverola, Emily Kubin, Anton Kunnari, Yordan Kutiyski, Oscar Laguna, Josh Leota, Eva Lermer, Jonathan Levy, Neil Levy, Chunyun Li, Elizabeth U. Long, Chiara Longoni, Marina Maglić, Darragh McCashin, Alexander L. Metcalf, Igor Mikloušić, Soulaimane El Mimouni, Asako Miura, Juliana Molina-Paredes, César Monroy-Fonseca, Elena Morales-Marente, David Moreau, Rafał Muda, Annalisa Myer, Kyle Nash, Tarik Nesh-Nash, Jonas P. Nitschke, Matthew S. Nurse, Yohsuke Ohtsubo, Victoria Oldemburgo de Mello, Cathal O’Madagain, Michal Onderco, M. Soledad Palacios-Galvez, Jussi Palomäki, Yafeng Pan, Zsófia Papp, Philip Pärnamets, Mariola Paruzel-Czachura, Zoran Pavlović, César Payán-Gómez, Silva Perander, Michael Mark Pitman, Rajib Prasad, Joanna Pyrkosz-Pacyna, Steve Rathje, Ali Raza, Gabriel G. Rêgo, Kasey Rhee, Claire E. Robertson, Iván Rodríguez-Pascual, Teemu Saikkonen, Octavio Salvador-Ginez, Waldir M. Sampaio, Gaia C. Santi, Natalia Santiago-Tovar, David Savage, Julian A. Scheffer, Philipp Schönegger, David T. Schultner, Enid M. Schutte, Andy Scott, Madhavi Sharma, Pujan Sharma, Ahmed Skali, David Stadelmann, Clara Alexandra Stafford, Dragan Stanojević, Anna Stefaniak, Anni Sternisko, Augustin Stoica, Kristina K. Stoyanova, Brent Strickland, Jukka Sundvall, Jeffrey P. Thomas, Gustav Tinghög, Benno Torgler, Iris J. Traast, Raffaele Tucciarelli, Michael Tyrala, Nick D. Ungson, Mete S. Uysal, Paul A. M. Van Lange, Jan-Willem van Prooijen, Dirk van Rooy, Daniel Västfjäll, Peter Verkoeijen, Joana B. Vieira, Christian von Sikorski, Alexander Cameron Walker, Jennifer Watermeyer, Erik Wetter, Ashley Whillans, Robin Willardt, Michael J. A. Wohl, Adrian Dominik Wójcik, Kaidi Wu, Yuki Yamada, Onurcan Yilmaz, Kumar Yogeeswaran, Carolin-Theresa Ziemer, Rolf A. Zwaan, Paulo S. Boggio

**Affiliations:** 1 Department of Psychology and Neural Science, New York University, New York, NY USA; 2 School of Psychology, University of Kent, Canterbury, England; 3 Department of Economics, Middlesex University London, London, England; 4 Department of Strategy and Management, Norwegian School of Economics, Bergen, Norway; 5 SWPS University of Social Sciences and Humanities, Poznań, Poland; 6 Department of Psychological Sciences, College of William and Mary, Williamsburg, VA USA; 7 Institute of Social Sciences Ivo Pilar, Zagreb, Croatia; 8 Department of Philosophy, Macquarie University, Sydney, NSW Australia; 9 Stanford Graduate School of Business, Stanford University, Stanford, CA USA; 10 Institute of Communication Science, Friedrich-Schiller University Jena, Jena, Germany; 11 School of Human Sciences, Institute for Lifecourse Development, University of Greenwich, London, England; 12 Department of Experimental Psychology, University of Oxford, Oxford, England; 13 Center for Human Brain Health, School of Psychology, University of Birmingham, Birmingham, England; 14 Department of Psychology, Macquarie University, Sydney, NSW Australia; 15 KU Leuven, Leuven, Belgium; 16 National Research University Higher School of Economics (HSE), Moscow, Russia; 17 De La Salle University, Manila, Philippines; 18 Department of Psychology, University of the Witwatersrand, Johannesburg, South Africa; 19 Department of Political Science, Aarhus University, Aarhus, Denmark; 20 X University, Toronto, ON Canada; 21 Department of Government, Dartmouth College, Hanover, NH USA; 22 Department of Psychology, University of Toronto, Toronto, ON Canada; 23 Gatton College of Business and Economics, University of Kentucky, Lexington, KY USA; 24 Department of Mass Communication, National University of Science and Technology (NUST), Islamabad, Pakistan; 25 Department of Management, Aarhus University, Aarhus, Denmark; 26 Faculty of Social and Economic Sciences, Comenius University, Bratislava, Slovakia; 27 IntraMed, Buenos Aires, Argentina; 28 Macedonian Academy of Sciences and Arts, North Macedonia, Republic of North Macedonia; 29 University of Colorado Boulder, Boulder, CO USA; 30 ESEIS/COIDESO [ESEIS, Social Studies and Social Intervention Research Center; COIDESO, COIDESO, Center for Research in Contemporary Thought and Innovation for Social Development], University of Huelva, Huelva, Spain; 31 Faculty of Social Work, University of Huelva, Huelva, Spain; 32 WHU – Otto Beisheim School of Management, Vallendar, Germany; 33 Latin American Brain Health Institute (BrainLat), Adolfo Ibáñez University, Santiago, Chile; 34 Global Brain Health Institute, University of San Andrés, Buenos Aires, Argentina; 35 Department of Educational and Counselling Psychology, University of Dhaka, Dhaka, Bangladesh; 36 Psychology Department, The Hebrew University of Jerusalem, Jerusalem, Israel; 37 Institute of Psychology, Jagiellonian University, Kraków, Poland; 38 Institute of Security and Global Affairs, Leiden University, The Hague, Netherlands; 39 Erasmus School of Law, Erasmus University Rotterdam, Rotterdam, Netherlands; 40 Department of Political Science, Vrije University (VU) Amsterdam, Amsterdam, Netherlands; 41 Department of Digital Humanities, University of Helsinki, Helsinki, Finland; 42 Department of Cognition, Emotion, and Methods in Psychology, University of Vienna, Vienna, Austria; 43 School of Psychology, National Autonomous University of Mexico, Mexico City, Mexico; 44 Department of Economics, National Taiwan University, Taipei, Taiwan; 45 Center for Research in Econometric Theory and Applications, National Taiwan University, Taipei, Taiwan; 46 Tribhuvan University, Kirtipur, Nepal; 47 Department of Leadership and Organizational Behavior, BI Norwegian Business School, Oslo, Norway; 48 Institute of Security and Global Affairs, Leiden University, Leiden, Netherlands; 49 Faculty of Governance and Global Affairs, Leiden University, Leiden, Netherlands; 50 Department of Psychology, University of Limerick, Limerick, Ireland; 51 Department of Psychology, The University of Western Ontario, London, ON Canada; 52 Center for Advanced Hindsight, Duke University, Durham, NC USA; 53 Department of Clinical Neuroscience, Karolinska Institute, Solna, Sweden; 54 Department of Management, University of Agder, Kristiansand, Norway; 55 Institute of Retail Economics, Stockholm, Sweden; 56 Department of Psychology, Lehigh University, Bethlehem, PA USA; 57 Department of Psychology, Monk Prayogshala, Mumbai, India; 58 Medicine Faculty, Cooperative University of Colombia, Villavicencio, Colombia; 59 Department of Bioethics, El Bosque University, Bogotá, Colombia; 60 Faculty of Medicine, Pontifical Javeriana University, Bogotá, Colombia; 61 Department of Management, Technology, and Economics, ETH Zürich, Zürich, Switzerland; 62 Department of Psychiatry and Medical Psychology, Research Institute, Medical University of Plovdiv, Plovdiv, Bulgaria; 63 Humanities and Social Sciences, Indian Institute of Management, Indore, India; 64 Institute of Social Sciences, Belgrade, Serbia; 65 Department of Psychology, Royal Holloway, University of London, London, England; 66 Center for the Politics of Feelings, School of Advanced Study, University of London, London, England; 67 Department of Behavioral and Cognitive Sciences, Faculty of Humanities, Education and Social Sciences, University of Luxembourg, Luxembourg City, Luxembourg; 68 Department of Political Science, National Taiwan University, Taipei, Taiwan; 69 Faculty of Political Science, National School for Political Studies and Public Administration, Bucharest, Romania; 70 Department of Psychology, University of Latvia, Riga, Latvia; 71 Department of Psychology, Princeton University, Princeton, NJ USA; 72 Department of Psychology, Speech, and Hearing, University of Canterbury, Christchurch, New Zealand; 73 Department of Psychology and Neuroscience, Duke University, Durham, NC USA; 74 Department of Psychology, University of Virginia, Charlottesville, VA USA; 75 School of Economics and Management, Hebei University of Technology, Tianjin, PR China; 76 School of Collective Intelligence, Mohammed VI Polytechnic University, Ben Guerir, Morocco; 77 Institute for Research and Development-Kurdistan, Middle East, Iraq; 78 Impact For Development, North Africa, Morocco; 79 Department of Psychology, University of Amsterdam, Amsterdam, Netherlands; 80 Department of Psychology, Jose Rizal University, Mandaluyong, Philippines; 81 Department of Economics, Norwegian School of Economics, Bergen, Norway; 82 Telenor Research, Oslo, Norway; 83 Department of Philosophy, University Cheikh Anta Diop, Dakar, Senegal; 84 School of Medicine and Health Sciences, University of Rosario, Bogotá, Colombia; 85 Moral Psychology and Decision Sciences Research Incubator, University of Rosario, Bogotá, Colombia; 86 School of Psychological Sciences, University of Melbourne, Parkville, VIC Australia; 87 Department of Psychological and Social Sciences, Penn State Abington, Abington, PA USA; 88 Institute of Psychology, University of Wrocław, Wrocław, Poland; 89 IMT School for Advanced Studies Lucca, Lucca, Italy; 90 Department of Economics and Management, University of Florence, Florence, Italy; 91 Department of Management, London School of Economics and Political Science, London, England; 92 Toulouse Business School, University of Toulouse, Toulouse, France; 93 Social Policy Institute of the Ministry of Labor, Family and Social Affairs of the Slovak Republic, Bratislava, Slovakia; 94 Department of Sociology, Tilburg University, Tilburg, Netherlands; 95 Department of Psychology, Penn State University, University Park, PA USA; 96 Rock Ethics Institute, Penn State University, University Park, PA USA; 97 Department of Psychology, Federal University of Santa Catarina, Florianópolis, Brazil; 98 Department of Economics, University of Bologna, Bologna, Italy; 99 IUSS Cognitive Neuroscience (ICoN) Center, Institute for Advanced Study of Pavia, Pavia, Italy; 100 Cognitive Computational Neuroscience Research Unit, Neurological Institute Foundation Casimiro Mondino, Pavia, Italy; 101 School of Psychology, Shenzhen University, Shenzhen, PR China; 102 Darden School of Business, University of Virginia, Charlottesville, VA USA; 103 Institute for Advanced Study in Toulouse, Université Toulouse 1 Capitole, Toulouse, France; 104 Department of Brain and Behavioral Sciences, University of Pavia, Pavia, Italy; 105 Cracow University of Economics, Kraków, Poland; 106 UBC Sauder School of Business, University of British Columbia, Vancouver, BC Canada; 107 Psychology Department, University of California - Los Angeles, Los Angeles, CA USA; 108 Laboratory of Psychology: Cognition, Behavior, and Communication (LP3C), Rennes 2 University, Rennes, France; 109 Laboratory of Social and Cognitive Psychology, Clermont Auvergne University, CNRS, Clermont-Ferrand, France; 110 Cavite State University-General Trias City Campus, Cavite, Philippines; 111 School of Economics and Finance, Queensland University of Technology, Brisbane, QLD Australia; 112 Center for Behavioural Economics, Society and Technology, Queensland University of Technology, Brisbane, QLD Australia; 113 Crawford School of Public Policy, Australian National University, Canberra, ACT Australia; 114 CESifo, University of Munich, Munich, Germany; 115 Department of International Trade, Boğaziçi University, Istanbul, Turkey; 116 Kieskompas - Election Compass, Amsterdam, Netherlands; 117 Hult International Business School Dubai, Dubai, UAE; 118 Department of Social Policy and Intervention, University of Oxford, Oxford, England; 119 Department of Media and Communications, University of Sydney, Sydney, NSW Australia; 120 Department of Psychiatry, University of the Witwatersrand, Johannesburg, South Africa; 121 University of Bayreuth, Bayreuth, Germany; 122 Department of Psychology, University of Waterloo, Waterloo, ON Canada; 123 Department of Peace Studies, University of Bradford, Bradford, England; 124 Philosophy and Social Studies Department, Rethymno, Greece; 125 Department of Psychology and Neuroscience, University of North Carolina at Chapel Hill, Chapel Hill, NC USA; 126 Department of Economics, Koc University, Istanbul, Turkey; 127 Department of Media and Communication, City University of Hong Kong, Kowloon Tong, Hong Kong; 128 University of Regensburg, Regensburg, Germany; 129 FOM University of Applied Sciences, Essen, Germany; 130 Graduate School for Transnational Studies, Free University of Berlin, Berlin, Germany; 131 Department of Management and Engineering, Linköping University, Linköping, Sweden; 132 Department of Psychology, University of Koblenz-Landau, Landau, Germany; 133 Department of Psychology, University of Alberta, Edmonton, AB Canada; 134 LMU Center for Leadership and People Management, Ludwig Maximilian University of Munich, Munich, Germany; 135 Ansbach University for Applied Sciences, Ansbach, Germany; 136 Baruch Ivcher School of Psychology, Interdisciplinary Center Herzliya (IDC), Herzliya, Israel; 137 Department of Neuroscience and Biomedical Engineering, Aalto University, Espoo, Finland; 138 Questrom School of Business, Boston University, Boston, MA USA; 139 School of Psychology, Dublin City University, Dublin, Ireland; 140 University of Montana, Missoula, MT USA; 141 Graduate School of Human Sciences Human Sciences, Osaka University, Suita, Japan; 142 SEELE Neuroscience, Mexico City, Mexico; 143 School of Psychology, University of Auckland, Auckland, New Zealand; 144 Faculty of Economics, Maria Curie-Skłodowska University, Lublin, Poland; 145 Department of Psychology, The City University of New York (CUNY) Graduate Center, New York, NY USA; 146 Australian National Centre for the Public Awareness of Science, Australian National University, Canberra, ACT Australia; 147 Department of Social Psychology, Graduate School of Humanities and Sociology, University of Tokyo, Tokyo, Japan; 148 Department of Public Administration and Sociology, Erasmus University Rotterdam, Rotterdam, Netherlands; 149 Center for Social Sciences, Hungarian Academy of Sciences Center of Excellence, Budapest, Hungary; 150 Institute of Psychology, University of Silesia, Katowice, Poland; 151 Department of Psychology, University of Belgrade, Belgrade, Serbia; 152 Department of Biology, Faculty of Natural Sciences, Universidad del Rosario, Bogotá, Colombia; 153 Vidyasagar College For Women, Kolkata, India; 154 AGH University of Science and Technology, Kraków, Poland; 155 Department of Psychology, University of Cambridge, Cambridge, England; 156 Department of Computer Science, University of Colorado Boulder, Boulder, CO USA; 157 Institute of Cognitive Science, University of Colorado Boulder, Boulder, CO USA; 158 Social and Cognitive Neuroscience Laboratory, Mackenzie Presbyterian University, São Paulo, Brazil; 159 Stanford University, Stanford, CA USA; 160 Department of Biology, University of Turku, Turku, Finland; 161 Cooperative University of Colombia, Bogotá, Colombia; 162 Newcastle Business School, University of Newcastle, Callaghan, NSW Australia; 163 Department of Philosophy, University of St Andrews, St Andrews, Scotland; 164 School of Economics and Finance, University of St Andrews, St Andrews, Scotland; 165 Department of Global Economics and Management, University of Groningen, Groningen, Netherlands; 166 Brain and Mind Institute, University of Western Ontario, London, ON Canada; 167 Western Interdisciplinary Research Building, University of Western Ontario, London, ON Canada; 168 Department of Sociology, University of Belgrade, Belgrade, Serbia; 169 Department of Psychology, Carleton University, Ottawa, ON Canada; 170 National University of Political Studies and Public Administration (SNSPA), Bucharest, Romania; 171 Research Institute at Medical University of Plovdiv), Division of Translational Neuroscience, Plovdiv, Bulgaria; 172 Department of Cognitive Science, ENS, EHESS, CNRS, Institut Jean Nicod, PSL Research University, Paris, France; 173 CREMA ‐ Center for Research in Economics, Management and the Arts, Basel, Switzerland; 174 The Warburg Institute, School of Advanced Study, University of London, London, England; 175 Institute of Cognitive Neuroscience, University College London, London, England; 176 Institute for Emerging Market Studies, The Hong Kong University of Science and Technology, Kowloon, Hong Kong; 177 Department of Psychology, Susquehanna University, Selinsgrove, PA USA; 178 Psychology Department, Dokuz Eylül University, İzmir, Turkey; 179 Department of Experimental and Applied Psychology, VU Amsterdam, Amsterdam, Netherlands; 180 Research School of Psychology, Australian National University, Canberra, ACT Australia; 181 Department of Behavioural Sciences and Learning (IBL), Linköping University, Linköping, Sweden; 182 Department of Psychology, Education and Child Studies, Erasmus University Rotterdam, Rotterdam, Netherlands; 183 University of Koblenz-Landau, Landau, Germany; 184 Health Communication Research Unit, School of Human and Community Development, University of the Witwatersrand, Johannesburg, South Africa; 185 Department of Business Administration, Stockholm School of Economics, Stockholm, Sweden; 186 Harvard Business School, Harvard University, Cambridge, MA USA; 187 Nicolaus Copernicus University, Toruń, Poland; 188 University of California, San Diego, La Jolla, CA USA; 189 Kyushu University, Fukuoka, Japan; 190 Department of Psychology, Kadir Has University, Istanbul, Turkey

**Keywords:** Viral infection, Human behaviour

Correction to: *Nature Communications* 10.1038/s41467-021-27668-9, published online 26 January 2022.

In this article the author name ‘Agustin Ibanez’ was incorrectly written as ‘Augustin Ibanez’. The original article has been corrected.

