# Supplementary material for: National identity predicts public health support during a global pandemic
Source: Nat Commun. 2022 Jan 26;13:517. doi: 10.1038/s41467-021-27668-9 (PMC8792004; doi:10.1038/s41467-021-27668-9)
Supplement: Supplementary file 1 — Supplementary Information [file 41467_2021_27668_MOESM1_ESM.pdf]

## Supplementary Information

### Study 1 Sample statistics

In multilevel analyses, such as ours, the biggest concern is often the higher-level sample size[1]. Retaining more samples allowed us to include a large enough number of countries for multilevel analysis. However, this could have implications for the precision of the coefficients in each nation so we have provided the sample size and other statistics from each nation. The confidence intervals for each country coefficient are available in **Supplementary Figures 1, 2,** and **3.**

**Supplementary Table 1:** *Summary of Samples. N stands for the sample size. Gender is coded as follows: 1 = male, 2 = female, 3 = other (0.3% of the total sample reported gender = 3). Standard deviations are reported in brackets. Cases/million and Deaths/million represent the number of cases (deaths) per million inhabitants at the start of data collection. Some countries have smaller sample sizes that fall short of n = 500; even so, we decided to include them in the analysis as MLM takes into account different numbers of observations.*

| Country | N | % female | Mean Age | Cases / million | Deaths / million |
|---|---|---|---|---|---|
| Argentina | 721 | 69.1 | 47 (15) | 89.97 | 4.42 |
| Australia | 2160 | 51.2 | 47 (18) | 268.95 | 3.32 |
| Austria | 1510 | 52.8 | 50 (14) | 1723.93 | 61.97 |
| Bangladesh | 553 | 54 | 32 (11) | 36.64 | 0.94 |
| Belgium | 1159 | 40.6 | 46 (19) | 4073.91 | 628.88 |
| Bolivia | 29 | 58.6 | 43 (13) | 89.34 | 4.67 |
| Brazil | 2076 | 59.5 | 37 (14) | 321.94 | 21.97 |
| Bulgaria | 666 | 65.1 | 31 (11) | 194.71 | 8.29 |
| Canada | 963 | 60.2 | 43 (17) | 1319.93 | 75.58 |
| Chile | 97 | 64.9 | 49 (15) | 737.48 | 10.57 |

| | | | | | |
|---|---|---|---|---|---|
| China | 1030 | 49 | 43 (14) | 70.16 | 3.47 |
| Colombia | 1274 | 62.6 | 41 (15) | 112.73 | 5.10 |
| Costa Rica | 25 | 36 | 45 (13) | 139.4 | 1.2 |
| Croatia | 515 | 52.1 | 46 (15) | 502.22 | 14.53 |
| Cuba | 43 | 51.2 | 49 (13) | 122.48 | 4.94 |
| Denmark | 566 | 49.5 | 49 (18) | 1531.15 | 73.49 |
| Dominican Republic | 36 | 80.6 | 40 (12) | 592 | 26.53 |
| Ecuador | 148 | 55.4 | 41 (12) | 1360.66 | 38.82 |
| El Salvador | 28 | 53.6 | 46 (12) | 50.31 | 38.82 |
| Finland | 689 | 46.3 | 39 (14) | 850.54 | 34.96 |
| France | 1119 | 54.7 | 43 (16) | 2477.06 | 348.16 |
| Germany | 1587 | 50 | 50 (16) | 1912.29 | 73.79 |
| Ghana | 389 | 32.8 | 31 (8) | 232.99 | 1.14 |
| Greece | 638 | 34.9 | 30 (11) | 236.38 | 12.69 |
| Guatemala | 48 | 43.8 | 45 (13) | 30.72 | 0.87 |
| Honduras | 24 | 70.8 | 39 (14) | 68.93 | 6.36 |
| Hungary | 506 | 51.6 | 49 (17) | 264.38 | 28.66 |
| India | 717 | 41 | 33 (12) | 21.77 | 0.69 |
| Iraq | 895 | 46.9 | 31 (14) | 48.06 | 2.29 |
| Ireland | 767 | 66.8 | 38 (15) | 4009.80 | 224.90 |

| | | | | | |
|---|---|---|---|---|---|
| Israel | 1253 | 50.8 | 41 (15) | 1751.69 | 22.97 |
| Italy | 1278 | 53.8 | 47 (17) | 3303.74 | 446.93 |
| Japan | 1212 | 50.8 | 47 (15) | 111.88 | 3.04 |
| Latvia | 1008 | 62.8 | 46 (14) | 426.04 | 6.77 |
| Macedonia | 710 | 55.6 | 38 (12) | 672.6 | 31.25 |
| Mexico | 1274 | 50.1 | 48 (14) | 123.05 | 11.36 |
| Morocco | 711 | 53 | 32 (13) | 114.35 | 4.5 |
| Nepal | 534 | 52.7 | 28 (8) | 1.85 | 0 |
| Netherlands | 1297 | 46.4 | 50 (17) | 2224.54 | 262.38 |
| New Zealand | 510 | 50.2 | 46 (18) | 301.02 | 3.89 |
| Nicaragua | 16 | 62.5 | 43 (15) | 2.01 | 0.46 |
| Nigeria | 594 | 49.7 | 32 (11) | 6.82 | 0.20 |
| Norway | 532 | 53.4 | 47 (17) | 1399.45 | 37.75 |
| Pakistan | 532 | 53.2 | 27 (8) | 65.57 | 1.38 |
| Panama | 18 | 66.7 | 44 (17) | 1440.43 | 39.95 |
| Paraguay | 16 | 87.5 | 39 (9) | 32.76 | 1.29 |
| Peru | 91 | 45.1 | 46 (14) | 897.12 | 24.44 |
| Philippines | 521 | 50.1 | 37 (12) | 72.89 | 4.79 |
| Poland | 1815 | 49.5 | 46 (17) | 313.46 | 14.8 |
| Romania | 1005 | 50.5 | 42 (14) | 584.18 | 33.02 |

| | | | | | |
|---|---|---|---|---|---|
| Russia | 558 | 52.7 | 45 (15) | 603.09 | 5.49 |
| Senegal | 397 | 36 | 35 (13) | 46.44 | 0.57 |
| Serbia | 1070 | 73.5 | 43 (12) | 949.86 | 17.91 |
| Singapore | 556 | 51.1 | 43 (14) | 2557.27 | 2.48 |
| Slovakia | 1265 | 50 | 44 (16) | 252.93 | 3.3 |
| South Africa | 909 | 74.1 | 40 (13) | 82.95 | 1.56 |
| South Korea | 529 | 46.9 | 42 (14) | 208.21 | 4.72 |
| Spain | 1090 | 32.8 | 46 (14) | 4887.56 | 501.09 |
| Sweden | 1568 | 40.5 | 53 (15) | 1850.05 | 222.29 |
| Switzerland | 1056 | 50.9 | 48 (17) | 3403.03 | 194.28 |
| Taiwan | 833 | 50.3 | 44 (13) | 18.04 | 0.25 |
| Turkey | 1454 | 51 | 37 (15) | 1369.04 | 35.37 |
| Ukraine | 577 | 52.5 | 37 (8) | 214.60 | 5.24 |
| United Kingdom | 550 | 50.9 | 46 (16) | 2375.81 | 317.43 |
| United States | 1506 | 51.3 | 44 (17) | 3010.96 | 171.42 |
| Uruguay | 49 | 69.4 | 53 (14) | 179.71 | 4.34 |
| Venezuela | 96 | 56.3 | 47 (13) | 11.4 | 0.35 |

**Details about the Study 1 survey**

Participants took the following scales in random order. To obtain the complete dataset with all measures see ref. 2; https://osf.io/tfsza):

- A *limiting physical contact* scale, consisting of five items, as, for example, “During the days of the coronavirus (COVID-19) pandemic, I have been staying at home as much as practically possible”.
- A *physical hygiene* scale, consisting of five items, as, for example, “During the days of the coronavirus (COVID-19) pandemic, I have been washing my hands longer than usual”.
- A *policy support* scale, consisting of five items, as, for example, “During the days of the coronavirus (COVID-19) pandemic, I have been in favor of closing all schools and universities”.
- A *generosity* measure, measuring the proportion of the daily wage in the corresponding country a participant would keep for themselves vs. give to a national charity vs. give to an international charity[3].
- A two-item *psychological well-being* scale[4].
- A three-item *collective narcissism* scale[5].
- A two-item *national identification* scale (one item from ref. 6 and additional item measuring identity centrality).
- A *COVID-19 conspiracy beliefs* scale, consisting of four items such as “The coronavirus (COVID-19) is a bioweapon engineered by scientists.”
- A six-item *open mindedness* scale[7].
- A seven-item *morality-as-cooperation* scale[8].
- A two-item *trait optimism* scale[9].
- A four-item *social belonging* scale[10].
- A four-item *trait self-control* scale[11].
- A one-item *self esteem* scale[12].
- A six-item *narcissism* scale[13].
- A ten-item *moral identity* scale[14].
- A *COVID-19 risk perception* scale, consisting of two items such as “By April 30, 2021: How likely do you think it is that you will get infected by the Coronavirus (Covid-19)?” Available answers from 0% to 100%, with 10% increments.
- A one-item *political orientation* scale. Participants were asked: “Overall, what would be the best description of your political views?”. Available answers from 0 = very left leaning to 10 = very right leaning.
- A *moral cir*cle measure[15].
- A subjective physical health measure. Participants were asked: “In general, how would you rate your physical health as it is today?”. Available answers from 0 = “extremely bad” to 10 = “extremely good”.

After these scales, participants took a three-item *cognitive reflection test* (CRT). The test was a reworded version of the test proposed by Frederick[16]; we reworded the items because the classic CRT is very well known. After the CRT, participants answered some demographic questions.

**Study 1:**

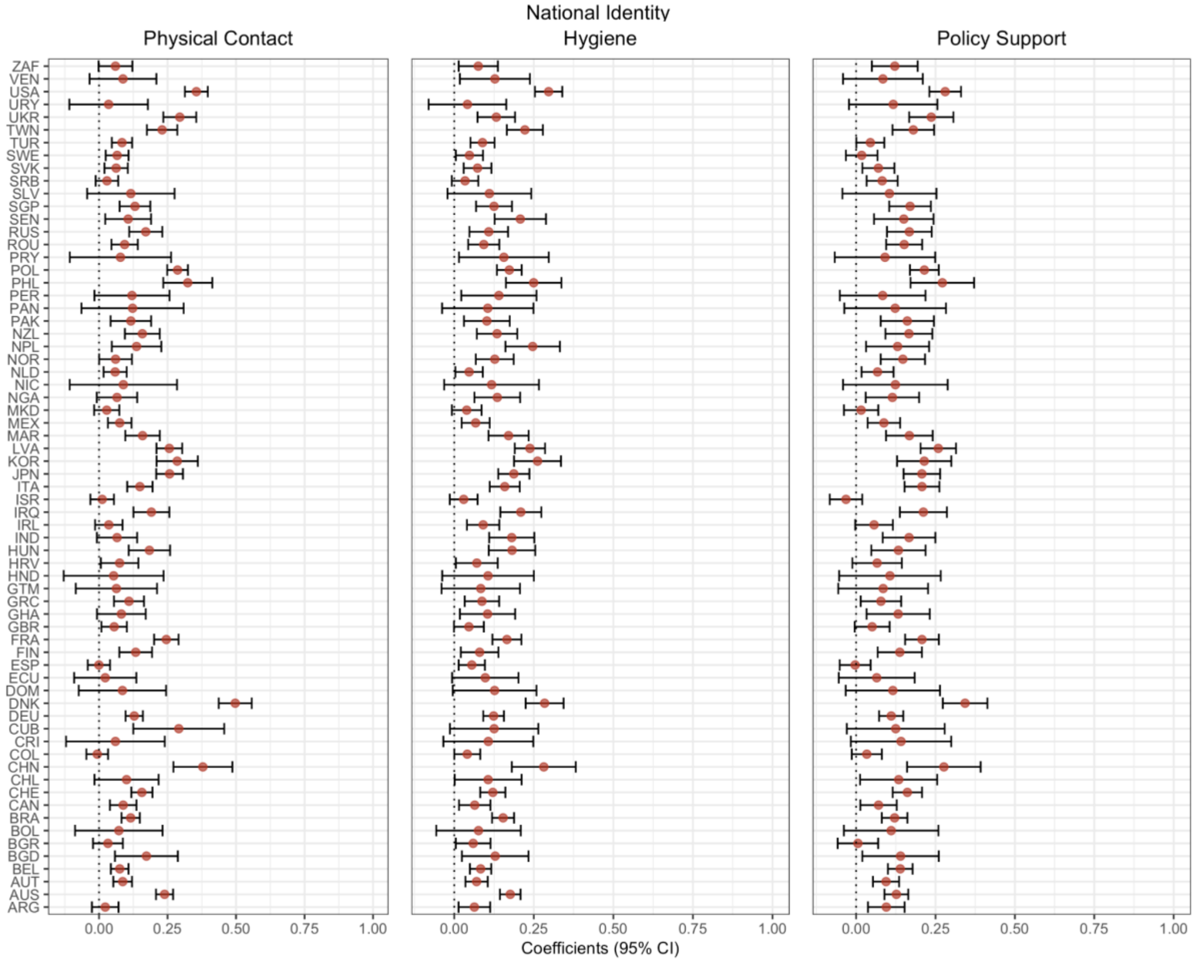


***Supplementary Figure 1.*** *Relation between national identification and public health measures in 67 countries and territories. The coefficients reflecting the relation between national identity and each of the health measures are presented for each country. The relation with physical contact (left), hygiene (center), and policy support (right) are presented with 95% confidence intervals.*

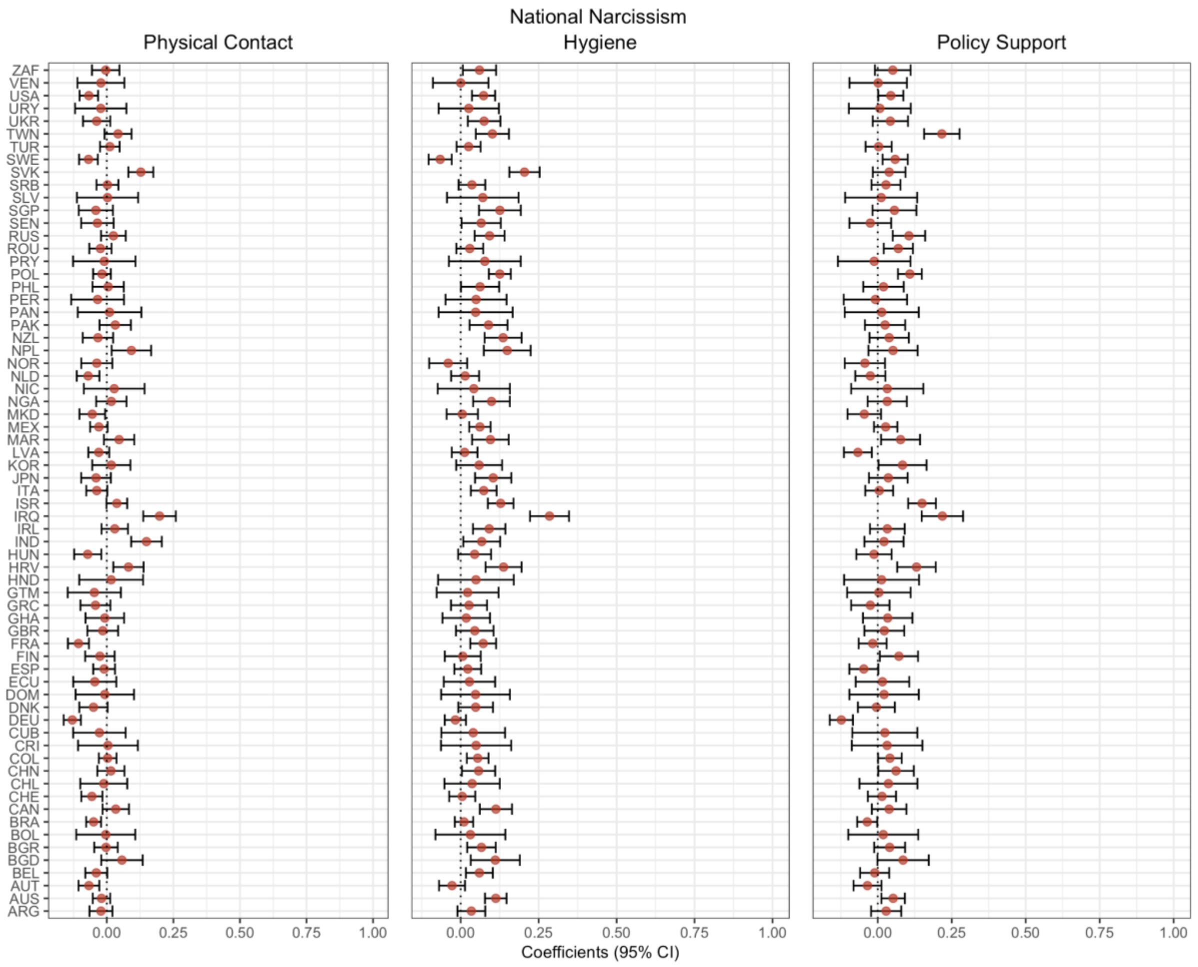


***Supplementary Figure 2.*** *Relation between national narcissism and public health measures in 67 countries and territories. The coefficients reflecting the relation between national narcissism and each of the health measures are presented for each country. The relation with physical contact (left), hygiene (center), and policy support (right) are presented with 95% confidence intervals.*

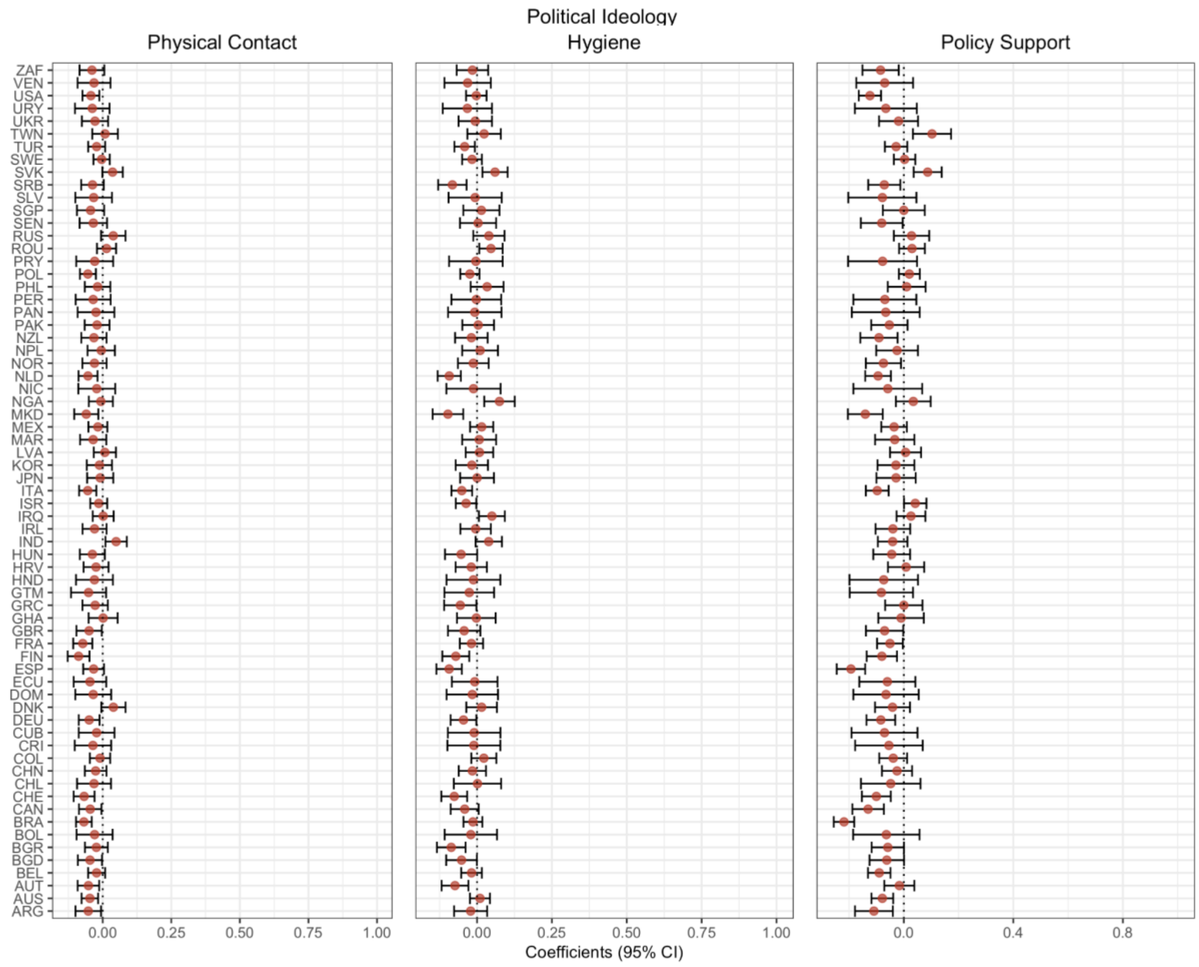

***Supplementary Figure 3.*** *Relation between political ideology and public health measures in 67 countries and territories. The coefficients reflecting the relation between political ideology and each of the health measures are presented for each country. The relation with physical contact (left), hygiene (center), and policy support (right) are presented with 95% confidence intervals.*

**Study 1: Differences as a function of sample characteristics and reliability**

We collected data in 67 countries. In 28 of these countries, we were able to obtain representative samples in terms of sex, age, and education. We collected convenience samples in 36 countries, and in three countries the sampling was mixed. We examined possible differences between countries as function of the representativeness of the sample by including a contrast coded variable (1 = representative, 0 – mixed, -1 = non-representative) at the country-level in the models that examined relationships between our three public health measures (spatial distancing, physical hygiene, and policy support) and our three predictor variables (national narcissism, national identification, and political ideology).

We found that means for the three public health measures were higher in non-representative samples than they were in representative samples. We found one significant moderating effect for slopes ($g_{021}$ = .038, $t$ = 2.55, $p$ = .014). In the analysis of spatial distancing, the slope for national identification was weaker for countries that had obtained non-representative samples than it was for countries that were able to obtain representative samples. Although it was slightly weaker, we should note that the slope for countries with non-representative samples (.08) was still significantly different from 0 ($p$ < .001).

Although our analyses accounted for individual differences in the reliability of our outcomes and the unreliability of slopes, to examine more thoroughly if unreliability may have confounded our results, we conducted a series of analyses in which we examined if the coefficients (slopes and intercept) varied as a function of whether the measures in an analysis were reliable or not. Following the guidelines suggested by Shrout[17], we defined reliable as .6 or above.

For the analyses of spatial distancing, the outcome and predictors were reliable for 50 countries, for physical hygiene, they were reliable for 53 countries, and for policy support they were reliable for 57 countries. Similar to how we examined the possible influence of the representativeness of the sample, we added a contrast coded variable (1 = reliable, -1 = not reliable) at the country-level in the models that examined relationships between our three outcomes and three predictors. These analyses found no significant effects for the reliability of our measures for intercepts or slopes.

**Study 1: HDI Results**

For all measures, except political ideology, there were negative relations between HDI scores and country-level means (see **Supplementary Table 2**). In other words, citizens in countries with higher scores on the global Human Development Index also reported less support for two of our COVID-19 public health measures. However, our dataset includes data from very few African countries, many of which have relatively low HDI scores but seem thus far to have fared better in the pandemic than higher-HDI countries (see ref. 18).[1]

***Supplementary Table 2**: Relationships between HDI (Human Development Index) scores and means of person-level variables. T-ratios and variance provide the test statistic and the percentage of variance explained for each variable.*

| ***Measure*** | ***HDI*** | ***t-ratio*** | ***Variance*** |
|---|---|---|---|
| *Spatial distancing* | *-.13* | *2.59* | *8%* |

[1] Although it is beyond the scope of the current paper, we think it would be interesting if future research examined whether people in lower and middle income countries indeed placed a greater faith in their political and medical institutions during the early stages of the pandemic, or whether they were motivated to simply report higher compliance with governmental guidelines.

| | | | |
|---|---|---|---|
| *Physical hygiene* | *-.40** | *4.98* | *34%* |
| *Policy support* | *-.59** | *5.81* | *37%* |
| *National narcissism* | *-.94** | *6.39* | *11%* |
| *National identity* | *-.52** | *7.33* | *31%* |
| *Political ideology* | *-.12* | *1.44* | *2%* |

Note: * $p < .001$

**Study 2: Correlations**

**Supplementary Table 3.** *Correlations between World Value Survey indices of national identification and each of the Google mobility indices (denoting a reduction in mobility in each category). The national identification score was produced by averaging national pride and national closeness and was correlated with a reduction in every measure of community mobility (rs = -.26 to -.44). The relationships were stronger for the item measuring national pride than country closeness.*

| | **National identification (average)** | **National pride** | **Country closeness** |
|---|---|---|---|
| **Mobility index (average)** | $-.40^{**}$ | $-.52^{***}$ | -.12 |
| **Retail and recreation** | $-.37^{*}$ | $-.46^{**}$ | -.12 |
| **Grocery and pharmacy** | $-.42^{**}$ | $-.54^{***}$ | -.13 |
| **Parks** | -.26 | $-.43^{**}$ | .02 |

| | | | |
|---|---|---|---|
| **Transit stations** | -.44** | -.48** | -.22 |
| **Workplaces** | -.37* | -.42** | -.17 |
| **Residential (reverse coded)** | -.29 | -.39* | -.07 |

*p*<.05, ***p*<.01, ****p*<.001

## Supplementary References


1. Maas, C. J. M., & Hox, J. J. Sufficient sample sizes for multilevel modeling. *Methodology* **1,** 86–92 (2005).

2. Azevedo, F., Pavlovic, T., Rêgo, G. G., Ceren, F. A., Gjoneska, B., Ross, R. M., Schoenegger, P., Riano, J., Cichocka, A., Capraro, V., Cian, L., Longoni, C., Chan, H. F., Gkinopoulos, T., Kantorowicz, J., ICSMP Consortium, Sampaio, W. Social and moral psychology of COVID-19 across 69 countries. https://osf.io/tfsza (2021).

3. Sjåstad, H. Short-sighted greed? Focusing on the future promotes reputation-based generosity. *Judgm.Decis. Mak.* **14,** 199-213 (2019).

4. Bjørnskov, C. How comparable are the Gallup World Poll life satisfaction data? *J. Happiness Stud.* **11,** 41-60 (2010).

5. Golec de Zavala, A., Cichocka, A., Eidelson, R., & Jayawickreme, N. Collective narcissism and its social consequences. *J. Pers. Soc. Psychol.* **97,** 1074–1096 (2009).

6. Postmes, T., Haslam, S. A., & Jans, L. A single-item measure of social identification: reliability, validity, and utility. *Br. J. Soc. Psychol.* **52,** 597–617 (2012).

7. Alfano, M., Iurino, K., Stey, P., Robinson, B., Christen, M., Yu, F., & Lapsley, D. Development and validation of a multi-dimensional measure of intellectual humility. *PLoS ONE* **12,** e0182950 (2017).

8. Curry, O. S., Chesters, M. J., & Van Lissa, C. J. Mapping morality with a compass: testing the theory of 'morality-as-cooperation' with a new questionnaire. *J. Res. Pers.* **78,** 106-124 (2019).

9. Scheier, M. F., Carver, C. S., & Bridges, M. W. Distinguishing optimism from neuroticism (and trait anxiety, self-mastery, and self-esteem): a re-evaluation of the Life Orientation Test. *J. Pers. Soc. Psychol*. **67,** 1063-1078 (1994).

10. Malone, G. P., Pillow, D. R., & Osman, A. The general belongingness scale (GBS): assessing achieved belongingness. *Pers. Individ. Differ.* **52,** 311-316 (2012).

11. Tangney, J. P., Boone, A. L., & Baumeister, R. F. High self-control predicts good adjustment, less pathology, better grades, and interpersonal success. In *Self-regulation and Self-control* 181-220. (Routledge, London, 2018).

12. Robins, R. W., Hendin, H. M., & Trzesniewski, K. H. Measuring global self-esteem: construct validation of a single-item measure and the Rosenberg self-esteem scale. *Pers. Soc. Psychol. Bull.* **27,** 151–161 (2001).

13. Back, M. D. et al. Narcissistic admiration and rivalry: disentangling the bright and dark sides of narcissism. *J. Pers. Soc. Psychol*. **105,** 1013-1037 (2013).

14. Aquino, K. & Reed, A. II. The self-importance of moral identity. *J. Pers. Soc. Psychol*. **83,** 1423-1440 (2002).

15. Waytz, A., Iyer, R., Young, L., Haidt, J. & Graham, J. Ideological differences in the expanse of the moral circle. *Nat. Commun.* **10,** 4389 (2019).

16. Frederick, S. Cognitive reflection and decision making. *J. Econ. Perspect.* **19,** 25-42 (2005).

17. Shrout, P. E. Measurement reliability and agreement in psychiatry. *Stat. Methods Med. Res*. **7,** 301–317 (1998).

18. Ghosh, D., Bernstein, J. A., & Mersha, T. B. COVID-19 pandemic: the African paradox. *J. Glob. Health* **10,** 020348 (2020).
