# Supplementary material for: National identity predicts public health support during a global pandemic
Source: Nat Commun. 2022 Jan 26;13:517. doi: 10.1038/s41467-021-27668-9 (PMC8792004; doi:10.1038/s41467-021-27668-9)
Supplement: Supplementary file 3 — Reporting Summary [file 41467_2021_27668_MOESM3_ESM.pdf]

nature portfolio

Corresponding author(s): Jay Van Bavel, jay.vanbavel@nyu.edu

Last updated by author(s): Aug 30, 2021

Reporting Summary

Nature Portfolio wishes to improve the reproducibility of the work that we publish. This form provides structure for consistency and transparency in reporting. For further information on Nature Portfolio policies, see our Editorial Policies and the Editorial Policy Checklist.

Statistics

For all statistical analyses, confirm that the following items are present in the figure legend, table legend, main text, or Methods section.

n/a	Confirmed	
☐	☒	The exact sample size (*n*) for each experimental group/condition, given as a discrete number and unit of measurement
☐	☒	A statement on whether measurements were taken from distinct samples or whether the same sample was measured repeatedly
☐	☒	The statistical test(s) used AND whether they are one- or two-sided<br>*Only common tests should be described solely by name; describe more complex techniques in the Methods section.*
☐	☒	A description of all covariates tested
☐	☒	A description of any assumptions or corrections, such as tests of normality and adjustment for multiple comparisons
☐	☒	A full description of the statistical parameters including central tendency (e.g. means) or other basic estimates (e.g. regression coefficient) AND variation (e.g. standard deviation) or associated estimates of uncertainty (e.g. confidence intervals)
☐	☒	For null hypothesis testing, the test statistic (e.g. F, t, r) with confidence intervals, effect sizes, degrees of freedom and P value noted<br>*Give P values as exact values whenever suitable.*
☒	☐	For Bayesian analysis, information on the choice of priors and Markov chain Monte Carlo settings
☐	☒	For hierarchical and complex designs, identification of the appropriate level for tests and full reporting of outcomes
☐	☒	Estimates of effect sizes (e.g. Cohen's d, Pearson's r), indicating how they were calculated

Our web collection on statistics for biologists contains articles on many of the points above.

Software and code

Policy information about availability of computer code

Data collection	The base questionnaire was created in Qualtrics - a survey software tool.
Data analysis	Study 1 was analyzed with HLM6 and HLM7 (Raudenbush et al., 2011; see Nezlek, 2010, for a description of using MLM to analyze data from multinational studies). Study 2 was analyzed with IBM SPSS Statistics v27.

For manuscripts utilizing custom algorithms or software that are central to the research but not yet described in published literature, software must be made available to editors and reviewers. We strongly encourage code deposition in a community repository (e.g. GitHub). See the Nature Portfolio guidelines for submitting code & software for further information.

Data

Policy information about availability of data

All manuscripts must include a data availability statement. This statement should provide the following information, where applicable:

- Accession codes, unique identifiers, or web links for publicly available datasets
- A description of any restrictions on data availability
- For clinical datasets or third party data, please ensure that the statement adheres to our policy

All reported materials and data are available at: https://osf.io/y7ckt/

Field-specific reporting

Please select the one below that is the best fit for your research. If you are not sure, read the appropriate sections before making your selection.

☐ Life sciences ☒ Behavioural & social sciences ☐ Ecological, evolutionary & environmental sciences

For a reference copy of the document with all sections, see nature.com/documents/nr-reporting-summary-flat.pdf

Behavioural & social sciences study design

All studies must disclose on these points even when the disclosure is negative.

Study description	Both studies are quantitative.
Research sample	Study 1: N=49,968 (Mean age = 43; Gender = 52% females). Of the 67 countries in which data were collected, representative samples were collected in 28, convenience samples were collected in 36, and both types of sampling were used in three countries.<br>Study 2: We accessed data from two publicly available datasets: the World Values Survey (Haerpfer et al., 2020) and the COVID-19 Google Community Mobility Reports which indicate how people's physical movement has changed in response to COVID-19 policies (available at www.google.com/covid19/mobility/).
Sampling strategy	Study 1: In April 2020, we launched a call using social media to collect data all over the world on psychological factors that might be related to COVID-19 pandemic response, with public health support as the primary outcome. Each team was asked to collect data from at least 500 participants, representative with respect to gender and age, in their own country or territory. Of the 67 countries in which data were collected, representative samples were collected in 28, convenience samples were collected in 36, and both types of sampling were used in three countries. Some countries have smaller sample sizes that fall short of n = 500; even so, we decided to include them in the analysis as MLM takes into account different numbers of observations.<br>Study 2: We analysed all 42 countries in which aggregate data was publicly available for both for national identification and mobility scores.
Data collection	We created a survey in English in the software Qualtrics that we sent to each team. Where necessary, each team translated the survey into the local language, using the standard forward-backward translation method, and then collected the data online (either via a professional data collection company or by snowball sampling on social media).
Timing	Most samples in Study 1 were collected in April and May 2020. Study 2 relied on existing data, but for mobility scores we included change in mobility in response to COVID-19 restrictions during April and May 2020, to match the period of Study 1 data collection.
Data exclusions	Study 1: Raw data we obtained from all collaborators were cleaned to exclude any duplicate answers as well as those younger than 18 years or older than 100 years. We then excluded data from two participants from Puerto Rico and 313 participants recruited from the UEA where it was difficult to establish participant nationality. This resulted in a sample of 51,089 participants. For the current analysis, we also excluded participants who had missing data on all six key variables of interest. We were left with a sample of 49,968 for analyses.<br>Study 2: We analysed all 42 countries in which aggregate data was publicly available for both for national identification and mobility scores.
Non-participation	We did not record response rates for each country.
Randomization	Participants were not allocated to experimental groups.

Reporting for specific materials, systems and methods

We require information from authors about some types of materials, experimental systems and methods used in many studies. Here, indicate whether each material, system or method listed is relevant to your study. If you are not sure if a list item applies to your research, read the appropriate section before selecting a response.

Materials & experimental systems

n/a	Involved in the study	
☒	☐	Antibodies
☒	☐	Eukaryotic cell lines
☒	☐	Palaeontology and archaeology
☒	☐	Animals and other organisms
☐	☒	Human research participants
☒	☐	Clinical data
☒	☐	Dual use research of concern

Methods

n/a	Involved in the study	
☒	☐	ChIP-seq
☒	☐	Flow cytometry
☒	☐	MRI-based neuroimaging

Human research participants

Policy information about studies involving human research participants

Population characteristics	See above.
Recruitment	Recruitment differed slightly depending on sample (see information about sampling strategy above).
Ethics oversight	University of Kent, UK

Note that full information on the approval of the study protocol must also be provided in the manuscript.
